# The AMERIGO Lander and the Automatic Benthic Chamber (CBA): Two New Instruments to Measure Benthic Fluxes of Dissolved Chemical Species †

**DOI:** 10.3390/s19112632

**Published:** 2019-06-10

**Authors:** Federico Spagnoli, Pierluigi Penna, Giordano Giuliani, Luca Masini, Valter Martinotti

**Affiliations:** 1 Istituto per le Risorse Biologiche e le Biotecnologie Marine (IRBIM), Consiglio Nazionale delle Ricerche (CNR), Ancona 60125, Italy; pierluigi.penna@cnr.it (P.P.); giordano.giuliani@cnr.it (G.G.); 2 Institute for Microelectronics and Microsystems, Consiglio Nazionale delle Ricerche, Bologna 40129, Italy; masini@bo.imm.cnr.it; 3 Dipartimento Sviluppo sostenibile e Fonti Energetiche, RSE SpA, Milano 20134, Italy; valter.martinotti@rse-web.it

**Keywords:** lander, benthic chambers, benthic fluxes of dissolved chemical species, marine technology, marine instrumentation

## Abstract

Marine environments are currently subject to strong ecological pressure due to local and global anthropic stressors, such as pollutants and atmospheric inputs, which also cause ocean acidification and warming. These strains can result in biogeochemical cycle variations, environmental pollution, and changes in benthic-pelagic coupling processes. Two new devices, the Amerigo Lander and the Automatic Benthic Chamber (CBA), have been developed to measure the fluxes of dissolved chemical species between sediment and the water column, to assess the biogeochemical cycle and benthic-pelagic coupling alterations due to human activities. The Amerigo Lander can operate in shallow as well as deep water (up to 6000 m), whereas the CBA has been developed for the continental shelf (up to 200 m). The lander can also be used to deploy a range of instruments on the seafloor, to study the benthic ecosystems. The two devices have successfully been tested in a variety of research tasks and environmental impact assessments in shallow and deep waters. Their measured flux data show good agreement and are also consistent with previous data.

## 1. Introduction

Marine environments are affected by strong ecosystem stressors that include direct human activities (e.g., marine traffic, offshore activities, mining, coastal works) and inputs (e.g., dumping of solid waste on the seafloor, anthropic inputs transported by rivers, ballast water discharge) and chemical and climate changes that act on a global scale (e.g., raised CO_2_ levels and air temperature). These stressors affect marine chemistry and processes by inducing ocean acidification, global sea warming, and changes in hydrological and biogeochemical cycles [1]. Human activities also result in an increased supply of trophic substances, which, in some environmental settings such as shallow enclosed seas with low hydrodynamics, can lead to dystrophic crises [2,3,4]. These stressors also have the potential to alter the biogeochemical cycles of elements such as carbon, phosphorus, nitrogen, silicon, and metals, which can severely damage economic activities, such as fishing and tourism [4,5]. Moreover, the introduction and accumulation of heavy metals and organic substances (Polycyclic Aromatic Hydrocarbons (PAHs), pesticides, drugs) can induce strong pollution problems involving both the water column and sediment. These pollutants can modify or be incorporated in the food chains, damaging the ecosystem and heightening the risk for human health [5]. In particular, in the past few decades the marine biogeochemical cycle of carbon has undergone an acceleration as a consequence of increased atmospheric CO_2_, which has resulted in reduced seawater pH and carbon sinking rates [6]. 

The alterations in the marine biogeochemical cycles of elements and pollution due to human activities can affect the water column, the bottom sediment, and the transfer processes at the sediment-water interface. The research into and the development of devices to enhance the study of marine biogeochemical and benthic-pelagic coupling processes is therefore very useful [7,8,9], also in Italy [10,11,12,13].

A variety of devices have been developed to study benthic-pelagic coupling processes. In the past few decades, benthic landers with different setups have been developed for a wide range of purposes and their technological features and models have extensively been reviewed [14,15,16,17,18,19,20,21,22]. Benthic landers are equipped with diverse sensors and devices according to the tasks they are deployed to perform, including microprofilers, planar optodes, and digital cameras to study sediment-water interface properties; eddy correlation systems to measure the fluxes of dissolved chemical species in extensive areas; video cameras to investigate the deep sea biota; and oceanographic sensors (O_2_, pH, redox potential (Eh or ORP), optical turbidity, CTD, current meters, sediment traps) to study the water column [8,9,23,24,25,26,27,28]. In the past few years, other and much more complex and expensive devices have also been developed, such as landers for hadal environments [29,30,31,32,33], for multipurpose uses, and for transporting other mobile devices [34,35,36,37].

An important benthic-pelagic coupling process is the flux of dissolved chemical species at the sediment-water interface, generated by early diagenesis processes [38,39,40] or by volcanic benthic exhalation [41,42]. Such fluxes can strongly affect the chemistry of the water column, hence its ecology. This is especially true of shelf and coastal environments, where the high intensity of early diagenesis processes, due to a high reactive organic matter content in surface sediments, produces strong fluxes that affect shallow water columns [43].

The benthic fluxes of dissolved substances can be studied by onboard incubation or by in situ experiments, which are usually more reliable [44]. Fluxes are measured in benthic chambers handled by divers [45] in shallow waters, or mounted on benthic landers in deeper waters [44].

Landers equipped with one or more benthic chambers have been developed in the past [46,47,48,49,50,51,52,53,54,55]. 

Two new, low-cost, light, and easy to handle devices, the Amerigo Lander and the Automatic Benthic Chamber (CBA), which have recently been devised to study benthic ecosystems, particularly the fluxes of dissolved substance between sediment and the water column, are illustrated in this paper [56]. The two devices can also be employed jointly to improve the reliability of particular investigations.

The Amerigo Lander is basically a vector that can be deployed in shallow and deep bottoms and which returns to the surface at the end of its mission. It can carry a variety of instruments to study the water column and the benthic ecosystems by measuring various environmental parameters and processes. 

The Amerigo Lander and the CBA have provided the Italian scientific community with new and highly innovative instruments to investigate the benthic ecosystems and their interactions with the water column, bridging a scientific and technological gap with North American [25,35,37,46,47,48,49,50,51,52,53,54,55], Northern European [23,24,26,36,49] and Asian [27,28,29,30,33,34,50] countries. 

Their technical features make the two devices versatile and easy to use. The modularity of the lander, the various instruments that it can carry, and the much larger number of parameters that it can measure, compared with existing devices, make it a complete and original apparatus, suitable for operating both in shallows and at great depths. The most innovative features of the CBA are the larger area where measurements are acquired, which makes the fluxes more representative [23,24], and its easy and fast rigging, light weight, and maneuverability, which make it easy to use.

Both devices operate autonomously. Amerigo can work from shallow bottoms to depths of 6000 m, whereas the CBA has been designed for shallow water to shelf environments (up to about 200 m). Furthermore, the CBA can fit in the Amerigo, if required by research needs.

## 2. Amerigo Lander and Automatic Benthic Chamber: Technical Specifications and Equipment

### 2.1. The Amerigo Lander

**Figure 1:**
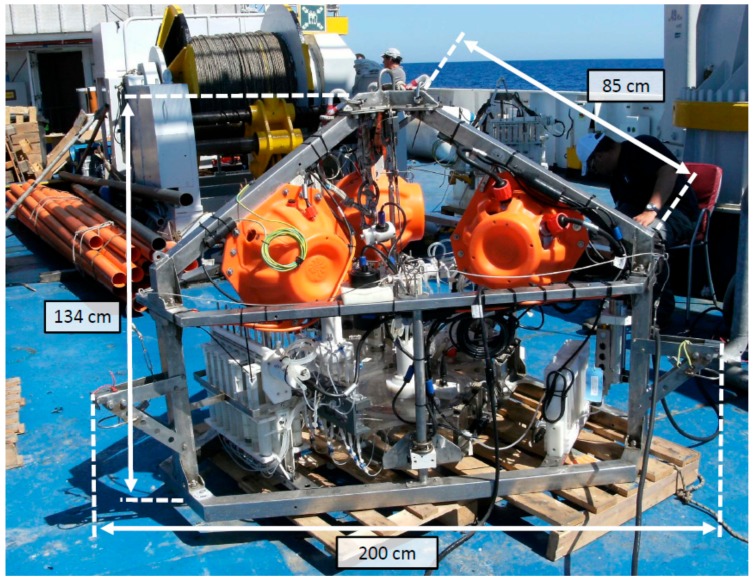
Photograph of the Amerigo Lander showing the tripod structure, the measurements, and the main instrumentation and devices.

The Amerigo Lander is essentially a carrier with a tripod structure that can host different types of instrumentation to measure biogeochemical and geophysical parameters and can collect water and sediment samples (Figure 1). It has been conceived as a simple, low-cost, and practical device that can be employed frequently in a variety of research tasks and environments, since it does not require huge resources, large vessels, or long missions. 

Since it reaches the bottom through a speed-controlled free-fall and returns to the surface by positive buoyancy, after a timed release of the ballast weights, it requires no drive or propulsion systems, such as thrusters or cables, nor divers for positioning and recovery. It is also completely automatic, because all hosted instruments and mechanical devices are powered in situ and activated and managed by electronics and software, again without the need for divers or cables. Obviating the need for divers also allows for overcoming stringent safety issues, particularly Italian safety regulations, with considerable savings. The elimination of a cable connection to the vessel makes the lander easy to handle, because the support ship can move while the lander is working on the seafloor. 

Another important advantage is its modular structure. In fact, the basic tripod structure can support a variety of components and instrumentation, which can be assembled and set to meet diverse research needs and environmental situations, ensuring flexible and simple operability. In particular, the lander’s electronics and power supply have been developed to enable management of additional devices and operations and for increased deployment time. Its modular structure allows the Amerigo Lander to operate in shallow waters (lagoons, estuaries, continental shelves) and deep-bottoms (abyssal plains). Further savings have been obtained from the electronic housings and the release mechanisms.

The lander’s basic structure consists of a stainless-steel tripod measuring 200 cm in width and 134 cm in height (Figure 1). All the electronic and mechanical devices required for reaching the seabed and returning to the surface and for hosting and managing the instruments operating on the seafloor are installed in the tripod (Figure 1). 

**Figure 2:**
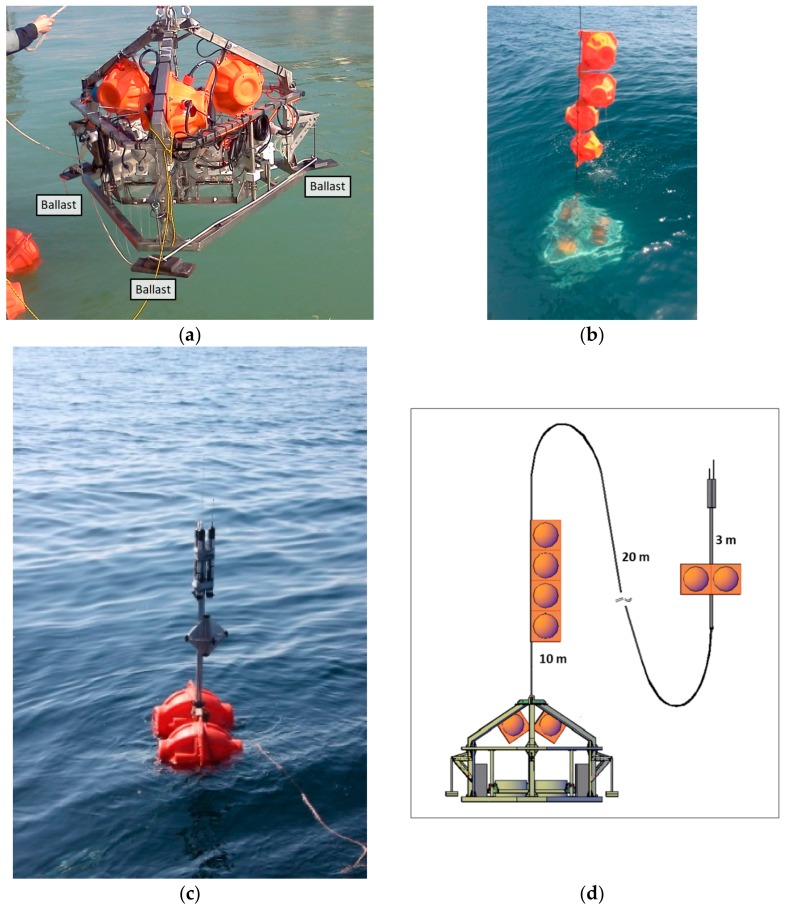
Amerigo Lander descent configuration. (**a**) The ballast weights mounted on the tripod; (**b**) the four buoys tethered to the tripod structure; (**c**) the recovery pole with the two buoys and the three localization devices; and (**d**) a schematic drawing of the Amerigo Lander during deployment on the seafloor.

The bottom is reached by a free fall without the need for thrusters or power, because the descent configuration envisages three ballast weights at the three ends and four buoys (Figure 2), which result in negative buoyancy.

The buoys are commercially available glass spheres (outer diameter 432 mm, thickness 14 mm, buoyancy 260 N), built for depths up to 6700 m (Nautilus Marine Service GMBH, VITROVEX Deep Sea Floatation Sphere), protected by a plastic shell (Nautilus Marine Service GMBH, SR330). The equilibrium between the buoys and the weights depends on the lander’s weight in the water, its descent and landing speed, and ascent requirements. 

In the present configuration, the lander weighs 294 kg in air and 131 kg in water and the 4 buoys have a buoyancy in seawater of 1040 kg, whereas the ballast weighs 45 kg (15 kg per weight). The buoys are tethered to the tripod with a rope 10 cm in thickness and 5 m in length (Nautilus Marine Service GMBH, EDDYROPE). Another rope, 10 cm in thickness, is tied to the recovery pole. The pole is fitted with 2 buoys the same size as the 4 buoyancy array buoys and with a 10 kg ballast weight, to support the recovery devices about 2 m above the sea surface (Figure 2d). 

**Figure 3:**
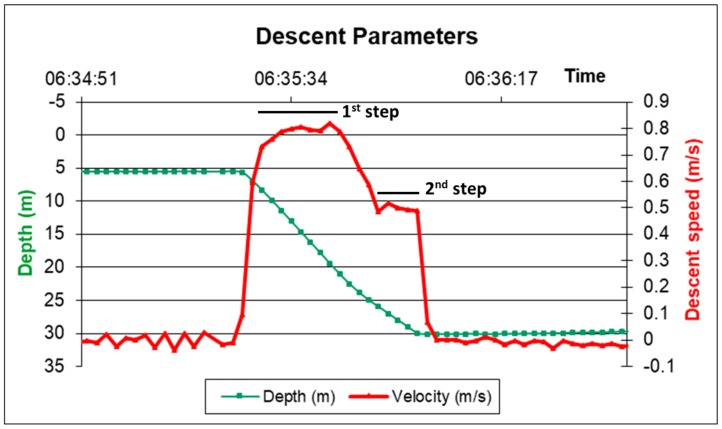
Amerigo Lander descent data. The data refer to the setup with three benthic chambers. Red line: Descent and landing speed. First step: Speed before activation of the first 2-buoy array; Second step: Speed after activation of the two buoys of the recovery pole. Green line: Depth profile.

With this configuration the lander’s initial descent speed is 0.78 m/s. The thrust of the 2 buoys tied to the recovery pole reduces the descent and landing speed to 0.52 m/s (Figure 3). With this set up, the lander can reach the bottom fast enough to avoid being shifted too much by lateral currents, and slowly enough to avoid an excessively strong impact.

The buoys are tethered to the tripod by a rope, not mounted directly on the tripod as in several other landers [26,47,50,51,54,55]. This solution has been adopted for two reasons, as follows: (i) As separation of the main tripod structure from the buoys makes the lander lighter and smaller, enhancing maneuverability in deployment and recovery operations, this solution requires smaller frames and less powerful winches; and (ii) this setup allows the lander to be transported in an ordinary van that can be driven with an ordinary license. Furthermore, the buoys and the recovery pole may not be needed in case of operation in shallow water, further enhancing maneuverability and reducing vessel size requirements. These features increase cost-effectiveness and the ease of organization. 

**Figure 4:**
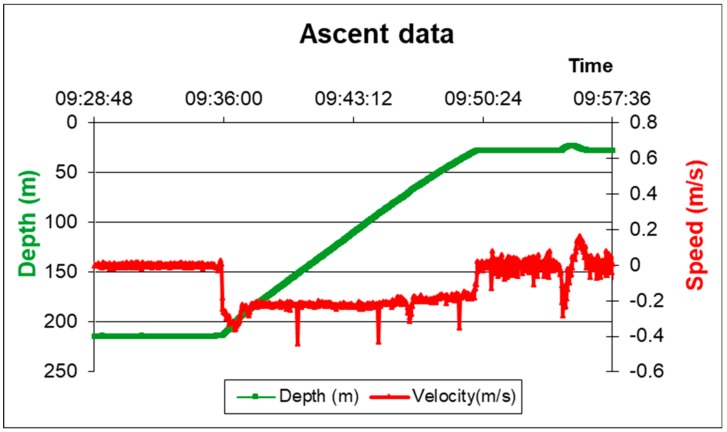
Amerigo Lander ascent data. The data refer to the setup with three benthic chambers. Red line: Ascent speed. Green line: Depth profile.

After completion of the measurement and sampling operations, the lander returns to the surface autonomously. The release of the three ballast weights and the thrust of the buoy array results in positive buoyancy. In the present configuration (4 thrust buoys, 2 recovery pole buoys, 3 benthic chambers) its ascent speed is about 0.2 m/s (Figure 4).

**Figure 5:**
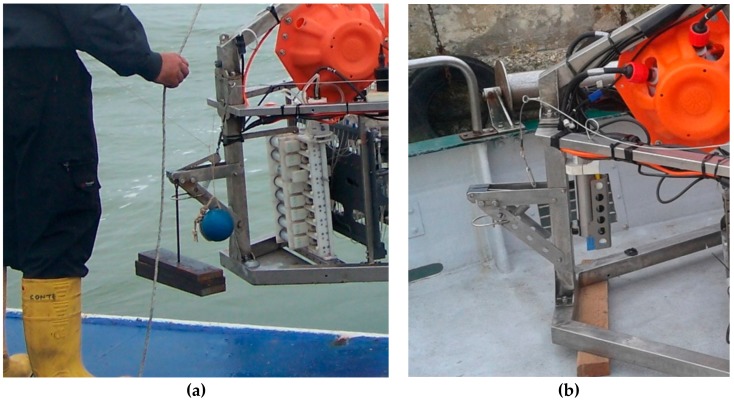
The three lever hooks in a locked (**a**) and unlocked (**b**) position.

At the end of the bottom operations, the ballast weights are released by a burn wire mechanism that unlocks the three lever hooks (Figure 5). This system is much less expensive than acoustic release [57] and further contributes to make the lander cost-effective and easy to use and to program.

**Figure 6:**
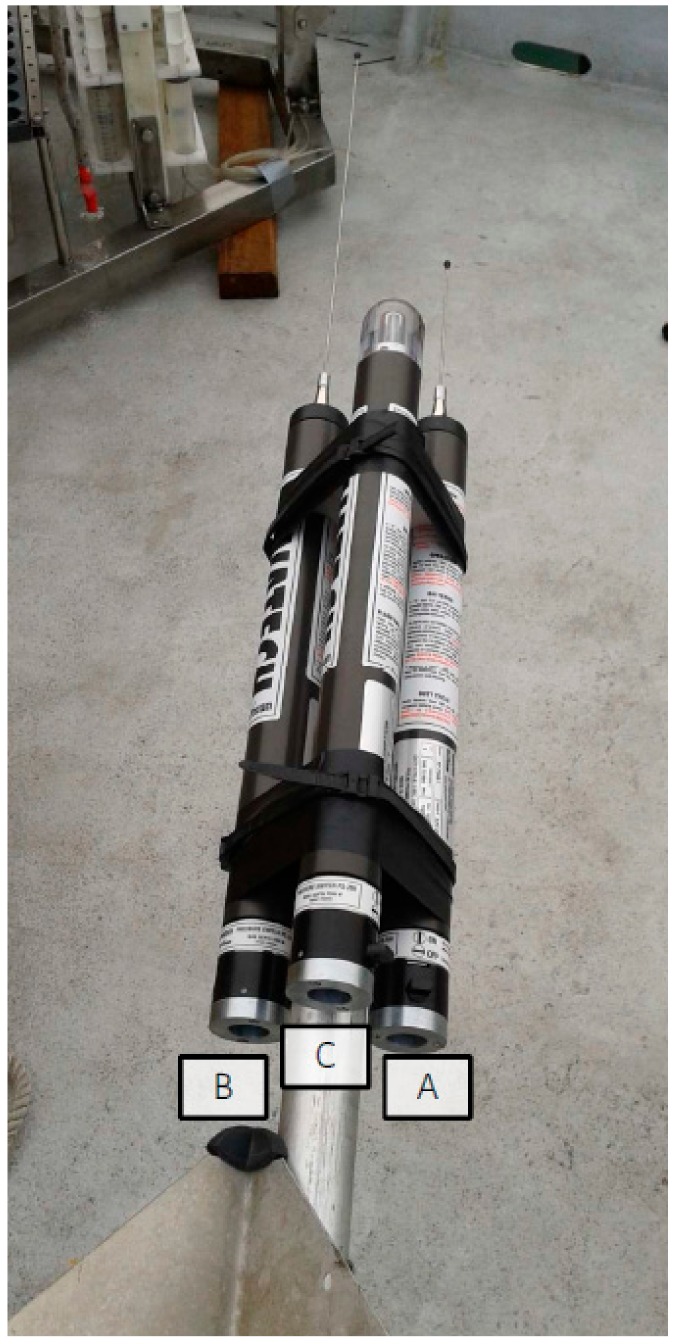
Amerigo Lander recovery system. (A) GPS localization (Novatech ARGOS Beacons); (B) directional radio localization system (Novatech Radio Beacon); and (C) flash for visual localization at night (Novatech Xenon Flasher).

In case of deployment in deep water, the Amerigo Lander may surface at a considerable distance from the dropping site, due to lateral sea currents during the ascent. To facilitate recovery, the lander is equipped with three redundant localization devices fitted on the top of the recovery pole, a GPS (Novatech ARGOS Beacons, Dartmouth, NS, Canada), a directional radio (Novatech Radio Beacon, Dartmouth, NS, Canada), and a flash (Novatech Xenon Flasher, Dartmouth, NS, Canada) for night recovery (Figure 6).

**Figure A1:**
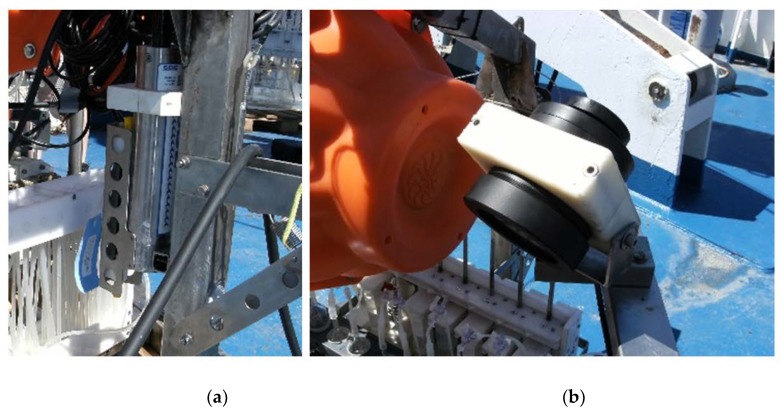
The Amerigo Lander monitoring instrumentation. (**a**) The SBE CTD mounted on the Amerigo tripod; (**b**) the Telesub Lanterna camera installed on the Amerigo Lander.

The Amerigo Lander is also equipped with instruments for monitoring and measuring the physical-chemical parameters throughout deployment, from descent to ascent. They include a CTD (SBE 37-SI MicroCAT, Sea-Bird Scientific, Bellevue, WA, USA) for continuous water column pressure, conductivity, and temperature recording, and a camera supporting an SD card from 4GB to 32GB (Telesub Lanterna, La Spezia, Italy) (Figure A1, Appendix A) for monitoring the lander’s operation. In fact, both instruments monitor the lander’s activities, particularly the beginning of descent, the descent speed, the landing, the functioning of the mechanical devices, the beginning and speed of the ascent, and the lander’s surfacing.

Additionally, in this case, both the CTD and the video camera are commercially available to save construction costs. In particular, the video camera is a commercially available camera hosted in a pressure-resistant case. 

### 2.2. Electronics and Power Supply

**Figure 7:**
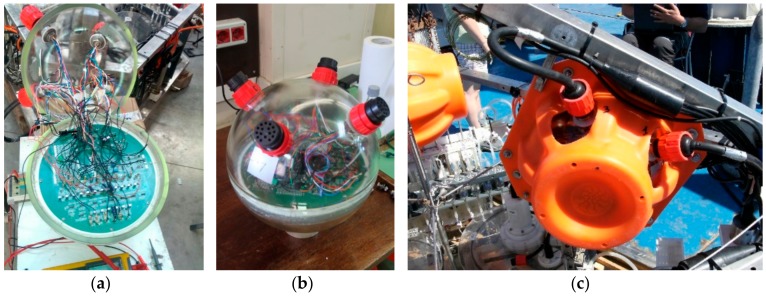
Amerigo Lander electronics: (**a**) Firmware testing phase and soldered cables inside the glass sphere; (**b**) electronics fitted in the glass sphere; (**c**) a glass sphere mounted on the tripod with the plastic case, marine connectors, and cables.

**Figure A2:**
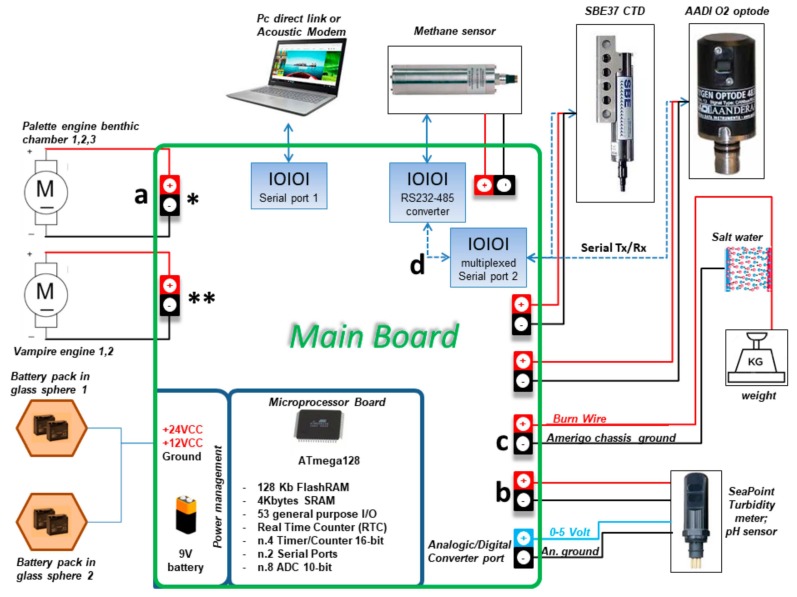
Electronics of the Amerigo Lander: Schematic drawing of the electronics diagram architecture. * Multiplied by three (palettes); ** multiplied by two (VAMPIRE motors); a: dedicated POWER-ON/OFF port (H bridge with electronic shunt); b: other “n” analogic devices; c: other “n” burn wire systems; d: other “n” digital sensors; blue dashed line: serial Tx/Rx port.

The Amerigo Lander’s electronics and power supply are developed in-house (Figure 7, Figure A2 of Appendix A). The system is available on request. The burn wire system and all the mechanical and electronic devices, sensors, and probes are powered, turned on, turned off, and managed by the electronics and the batteries fitted in the tripod. The data collected in situ are also stored in the Amerigo electronics. The main hardware components are configured as illustrated in Table A1 and Table A2, and Figure A2 of Appendix A. The philosophy of electronics is to be as open as possible, i.e., to enable fitting other sensors or devices by the availability of redundant on/off, serial, and analogic ports and the possibility of managing these sensors/devices and other operations by the software.

The Amerigo Lander is powered by two pairs of 12 V, 18 Ah, rechargeable lead batteries that are connected in parallel and fitted in a pressure-resistant case. The fact that they are commercially available and Pb-based involves lower managing and setup costs, compared to other metal-based batteries. The first pair is supported by the redundant second pair, which is only activated in case of exhaustion or failure of the first pair. This power supply supports about a 40 h operation of the lander in its present configuration. The Pb batteries can be increased or their type changed to support different configurations or to extend the operating time.

In the event of a failure of the general power supply or of the main electronics, a safety burn wire device powered by an independent 9 V battery and controlled by a dedicated electronic circuit is activated, after a predetermined time, to release the ballast for the final ascent. An additional safety system consists of a magnesium ring that is corroded by seawater. In case of the failure of all the electronic devices, its disappearance releases the ballast weights [58].

The electronics and the batteries are hosted in glass spheres built for depths of up to 7000 m (Nautilus Marine Service GMBH, VITROVEX Deep Sea Instrument Sphere; size: 13", outer/inner diameter: 330/306 mm, glass type: DURAN 8330), which are connected to the electronic devices, sensors, probes, and thrusters by marine connectors (Figure 7). The glass spheres are also commercially available and cost less than metal cylinders.

The two battery pairs are recharged on board by a cable that is removed before deployment. The cable is also used for serial port communication with the PC.

The main serial port (RS232–1) is devoted to communication with the PC. It allows for entering commands, changing the setup, downloading recorded data from the RAM flash memory or, in case of direct monitoring, it enables visualizing the data, the situation on the bottom, and the ongoing operations, as well as reporting malfunction alarms in real time (Figure A2 of Appendix A). The same port can be used to connect an acoustic modem for underwater communication.

The second serial port (RS232–2) is multiplexed in order to communicate with infinite RS-232 serial sensors, limited only by the hardware power connections (Figure A2 of Appendix A). 

Different systems have been designed to protect the lander’s electronics. The power supply to each electrical device (sensors, motors, batteries, burn wires) is protected by an electrical shunt that limits current drain. In case of failure of a device, the power supply to it is cut off to prevent a general electronic failure.

Furthermore, the open electronics and the excess of on/off, serial (by means of a multiplexed serial port), and analogic communication ports allows fitting many other electronic sensors, devices, and instruments, with respect to other system that are devised for standardization as well as useful use [28].

The following sensors are currently installed in the Amerigo Lander (Table 1) (Figure A2 of Appendix A):

An important question is the operation limit connected with temperature. The temperature operation limit of Amerigo coincides with the lower operation value between the sensors fitted in the benthic chambers and the polycarbonate (i.e., 40 °C (optode sensor); if the lander is used without sensors the temperature limit is <140 °C (polycarbonate)).

### 2.3. Burn Wire Device

The burn wire mechanism [51] consists of a metal wire coated with a plastic film that is corroded and then broken by an electric current at a bare point where the coating is interrupted (Figure A2, Appendix A). A simple 12 kg fishing wire coated with a thermo-shrinkable tube is a typical design. The plastic-coated wire usually keeps a lever hook locked. When the electric current runs through the wire, the bare point interacts with seawater, which triggers a reaction (1) that consumes the wire completely, leading to release of the lever hook, as follows:

(1) $$M_{solid}^{0}=M_{solute}^{2+}+2e^{-},$$

where *M*^0^ is the metal of the wire, 2*e*^−^ is the electric current, and *M*^2+^ is the metal in the solution. The electric circuit is closed by an electric mass on the metal tripod. By this method, any spring- or gravity-based mechanical device can be actioned by the release of a mechanical device or lever hook that can hold several tens of kilos, depending on the length of the lever (Figure 5). A metal wire, 0.4 mm in thickness with a resistance of a 12 Ω/m, exposed to an electric current of 200 mA in normal seawater (36 PSU) is usually consumed and broken in about 20 s. As mentioned above, the burn wire system is much less expensive than any acoustic release system.

### 2.4. Current Configuration of the Amerigo Lander

**Figure 8:**
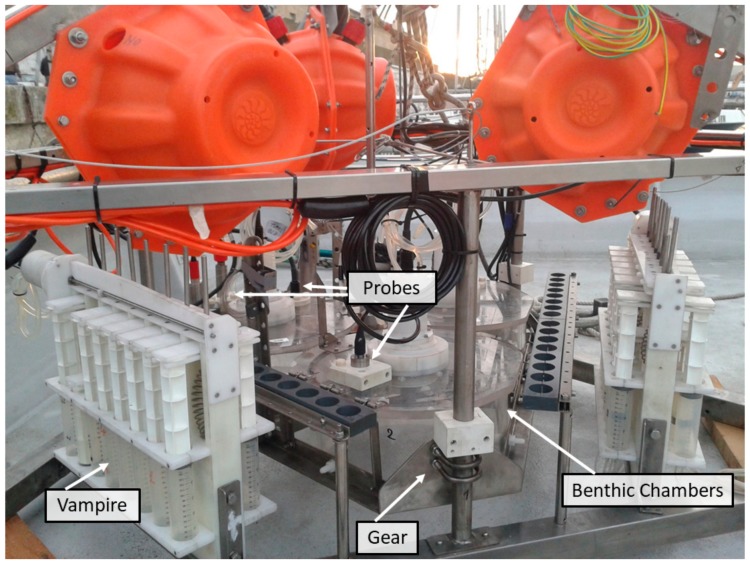
Amerigo Lander: The three benthic chambers, the two water sampling devices, the three probes in a chamber, and the chassis on which the chambers are mounted.

**Figure 9:**
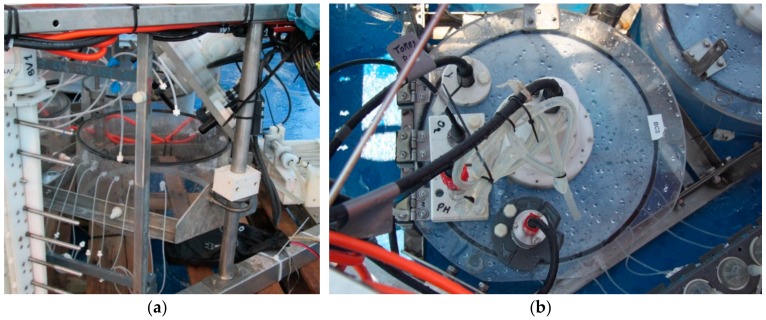
Amerigo Lander. (**a**) Lateral view of a benthic chamber mounted on the chassis with the top lid open, the tubes on the lateral wall to collect water samples, and the rotating paddle. (**b**) Top view of a benthic chamber with the closed top lid fitted with the oxygen, pH, methane, and turbidity sensor.

**Figure 10:**
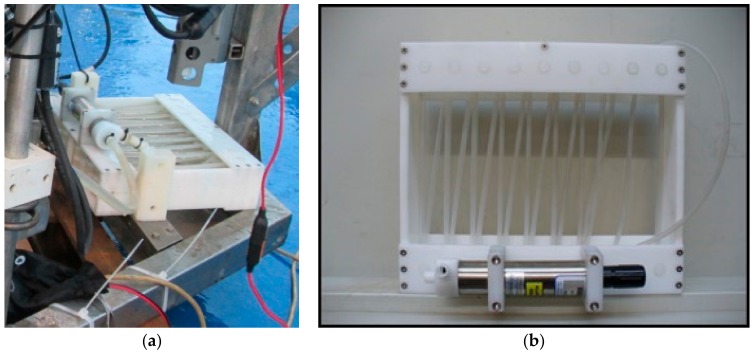
The OxyStat oxygen replacement device. (**a**) The pump and the tube connected to the interior of the chamber; (**b**) view from above.

**Figure 11:**
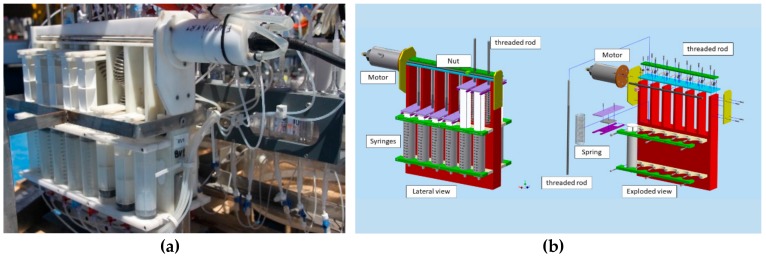
The VAMPIRE device allows drawing water into the chamber or expelling it as well as injecting a tracer inside the chamber. (**a**) Photograph of the VAMPIRE device installed on the Amerigo Lander; (**b**) schematic drawing of the VAMPIRE device.

**Figure 12:**
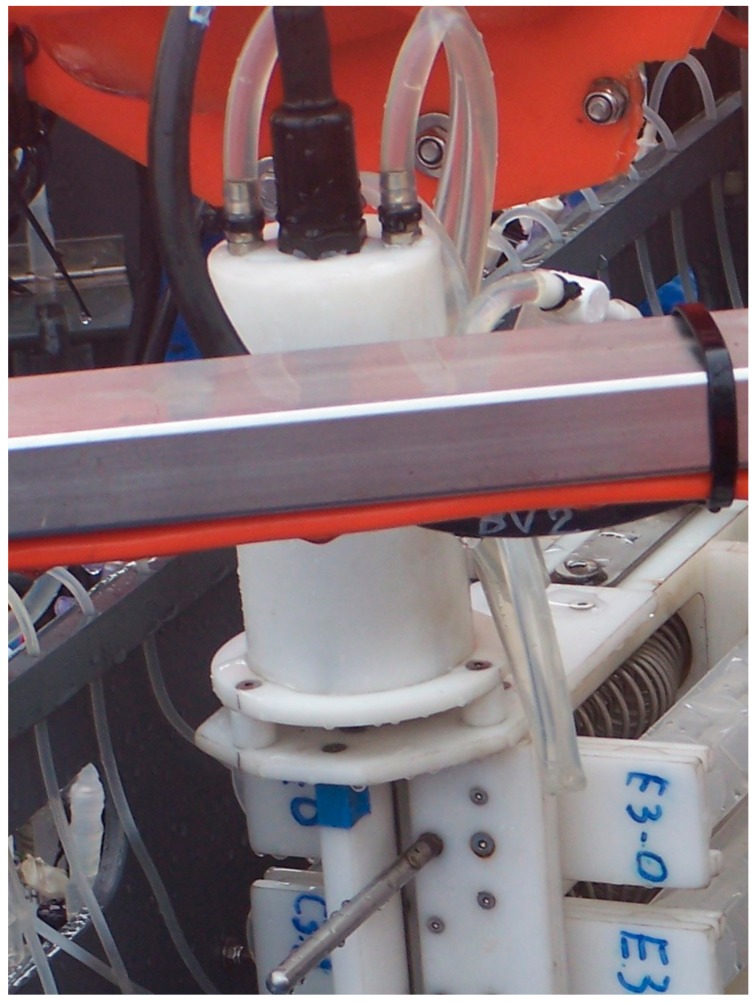
The Delrin case hosting the electric motors with the silicone tube pressure-compensation system.

In its current configuration, the Amerigo Lander is equipped with instruments and sensors for measuring benthic fluxes of dissolved chemical species and for monitoring physical-chemical parameters in the near-bottom sea water column. In particular, the former measurements at the sediment-water interface are performed using three benthic chambers, two water sampling systems, and some sensors fitted in the chambers (Figure 8, Figure 9, Figure 10, Figure 11 and Figure 12).

The benthic chambers are polycarbonate cylinders with a movable polycarbonate top lid (Figure 8 and Figure 9a). The cylinders measure 37 cm (inner diameter) by 20 cm (height) and have a countersink in the bottom to facilitate penetration in sediment [46].

The three benthic chambers are mounted on a chassis (Figure 8 and Figure 9a) that is released by a burn wire mechanism a few minutes after the tripod has landed on the seabed. The chambers are mounted 5 cm over the plate of the structure so that they can penetrate into the sediment for 5 cm, while the remaining 15 cm remain above the sediment. A few minutes after deployment of the chambers on the seabed, the lid of each chamber is unhooked, again by a burn wire device, thus closing the chamber and holding a known volume of water (approximately 17 L) overlying a known area of sediment [46]. The chassis and lid release time can be programmed by the lander’s software to adapt them to the research task and the type of sea bottom. 

The chemical and physical-chemical parameters in the chambers and some solute are measured by the following sensors fitted in each chamber during deployment: An oxygen sensor (AANDERAA, Oxygen Optode 3830, Aanderaa Data Instruments AS, Bergen, Norway), a turbidity sensor (Seapoint Turbidity Meter, Seapoint Sensors, Inc., Exeter, NH, USA), a methane sensor (ASD-Sensortechnik GmbH, METS methane sensor, Franatech Gmbh, Lüneburg, Germany) and a pH (AMT, pH-combined sensor, AMT Analysenmesstechnik GmbH, Rostock, Germany) sensor (Table 1) mounted on the lid (Figure 9b). The power-on/power-off and measurement intervals of each sensor can be set by the lander’s software according to research requirements.

The chambers also contain an OxyStat device, which allows for replacing the oxygen consumed in the chamber [59]. The device is connected to the chamber by a water pump (SEABIRD SBE5T, Sea-Bird Scientific, Bellevue, WA, USA) and a silicone tube (Figure 10) and is controlled by the lander’s software and the oxygen probe inside the chamber. In practice, the software receives the chamber oxygen concentration data and when its level falls below a given threshold, the software turns the OxyStat pump on. The oxygen-poor water in the chamber is pumped into the 15 m long gas-permeable silicone tube, it adsorbs oxygen from surrounding seawater, and is pumped back into the benthic chamber. Restoration of the oxygen level to the predetermined threshold results in the pump being turned off. The minimum and maximum oxygen concentrations can be set by the lander’s software before the mission or calculated on the basis of the initial concentration measured in the chamber.

Each benthic chamber is connected (Figure 9a) by silicone tubes (inner diameter, 1.5 mm, outer diameter 3 mm) to a water sampling device (VAMPIRE) to collect water or to introduce tracers into the chambers (Figure 11). The VAMPIRE consists of a Delrin frame hosting 8 pairs of syringes, each pair capable of collecting/injecting a maximum volume of 280 mL of water or tracer. If one syringe of the pair is not connected to the inside, seawater outside the chamber can be collected while the other syringe draws water inside the chamber. Each couple of syringes is activated by a nut moving on a rotating stainless-steel rod. The nut moves the levers that release the stainless-steel springs, which actuate the syringe pair in suction or injection mode. The rod is controlled by an electric motor (CBF Motors SRL, CRB35GM, CBF motors srl, Lissone, Italy) which is powered on and off by the lander’s software. Its timing and the activation of water sampling or tracer injection can also be set by the lander’s software.

**Figure A3:**
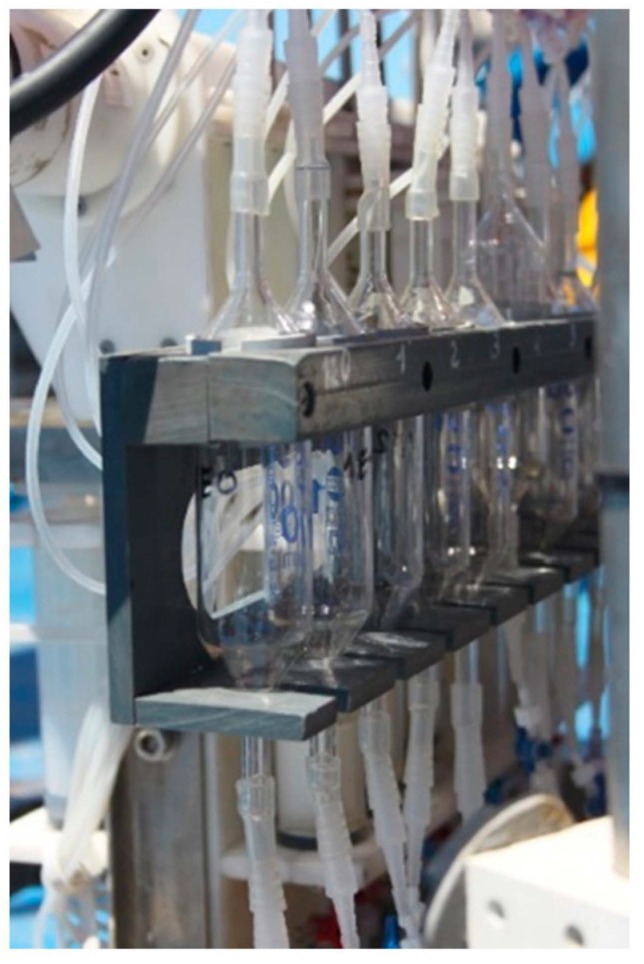
The set of gas-impermeable glass ampoules for water sampling for the analysis of dissolved gases.

In research tasks involving analysis of dissolved gases, a set of glass ampoules can be added before the syringes of the VAMPIRE device (Figure A3, Appendix A) to store the water samples in a gas-impermeable vessel until analysis. 

Each benthic chamber is also equipped with a stirring system, consisting of a rotating paddle mounted on the chamber lid (Figure 9a). The paddle is actioned by the coupling of an electric motor (CBF Motors SRL, CRB35GM, CBF motors srl, Lissone, Italy) to a permanent Neodymium magnet (Supermagnete, magnetic disk diameter 30 mm, height 15 mm, Neodymium, N42, nickel-plated, Webcraft GmbH, Gottmadingen, Deutschland). The paddle turns at a speed of 4–6 rpm, reproducing the hydrodynamics near the seabed, which is responsible of the formation of the benthic boundary diffusion layer at the sediment-water interface and, consequently, of the intensity of the benthic fluxes of dissolved chemical species. The motors of the rotating paddles are also activated by the lander’s software some minutes after closing of the lid.

Whereas the cases housing the electronics and the batteries are pressure-resistant, those housing the motors actuating the stainless-steel rod of the VAMPIRE device and the rotating paddles of the benthic chambers are pressure-compensated. These cases are Delrin cylinders with two silicone tubes (Figure 12) filled with a non-conductive liquid (commercially available Vaseline oil). If any air bubbles remain in the case, compression of the silicone tubes offsets the pressure difference between inside and outside, avoiding a collapse of the case.

At the end of each mission, i.e., after completion of the water sampling and sensor measurements in the benthic chambers, the three ballast weights are released by activation of the burn wire device, which induces positive buoyancy. The lander returns to the surface, where it is localized by means of the three positioning devices, and finally recovered on board. Missions typically last 8 to 36 h, depending on the tasks to be performed or the intensity of the benthic fluxes of dissolved substances, and are limited by the power supply, which, at present, supports the systems for about 40 h (see Section 2.2). However, as noted above, the open philosophy of the electronics allows the increasing of this time.

**Figure A4:**
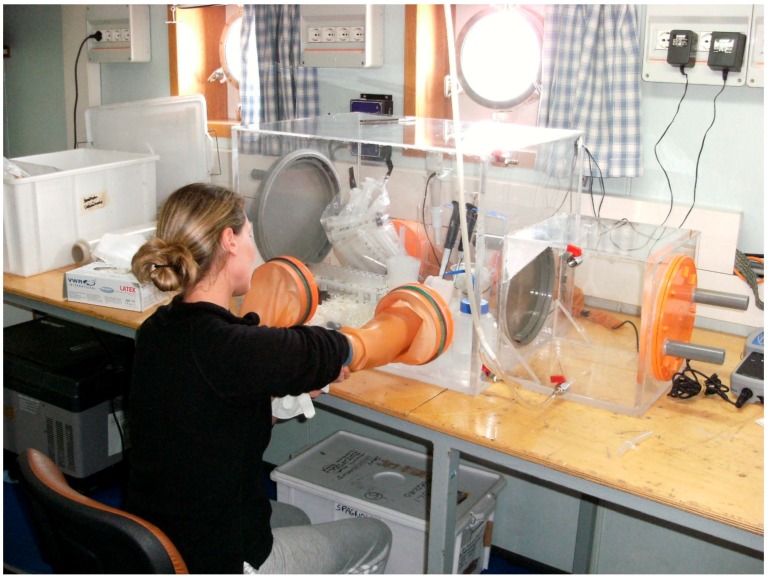
Filtering, subdivision into aliquots, and treatment of the water samples collected by the syringes of the Amerigo Lander is conducted in a Nitrogen glove-box.

After the recovery operations, the water samples taken by the syringes are collected from the VAMPIRE, divided and treated in an inert atmosphere (Figure A4, Appendix A) for immediate (on board) or subsequent (laboratory) chemical analysis of the solutes to be determined in the benthic fluxes. 

The data collected by the sensors are downloaded into a computer. 

The results of the chemical analyses and the data collected by the chemical sensor are then used to calculate the benthic fluxes of dissolved chemical species (see Section 3).

### 2.5. Typical Mission of the Amerigo Lander

The Amerigo Lander hardware is all managed by wizard software, developed in-house. The software helps the operator in setting all the parameters that are required for the lander’s function, to activate and test the motors, to monitor all the parameters in real time, to simulate a measurement mission, and to plan the activities for scheduling a mission. The software allows for the downloading and processing of the data collected during the mission and stored in situ. Finally, the software has been developed to fit further sensors and devices and to manage operations that are not currently scheduled.

**Figure 13:**
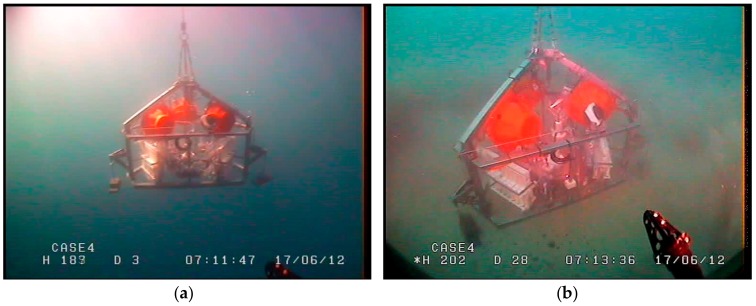

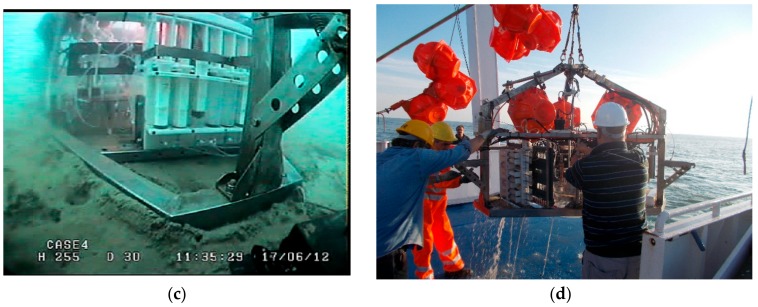
The Amerigo Lander photographed in some operational phases: (**a**) In the water before release; (**b**) on the seabed (view from above); (**c**) on the seabed (lateral view); and (**d**) on board after recovery.

A typical mission of the Amerigo Lander consists of six sequential phases that need to be correctly planned for the success of the mission, as follows (Figure 13):

1) After the on board programming and checks, such as control of the mooring line and testing of motors, sensors, communication, and security equipment, the lander is immersed into the sea, where it is held at a depth of 5 m, until activation of a dedicated burn wire mechanism releases a small buoy that confirms the functioning of the whole electronic system;
2) Following the buoy check, the lander is released for its free fall to the bottom;
3) After the lander has reached the seabed, a short interval is envisaged to allow the settling of the sediment resuspension, due to the impact of the tripod on the bottom, to settle;
4) Activation of a burn wire mechanism releases the chamber chassis, enabling its settling on the bottom and penetration into sediment for the first 5 cm; all the sensors in the chamber (oxygen, methane, turbidity) and CTD are sampled; the chambers are still open;
5) After another interval, to allow settling of the sediment resuspension due to the impact of the chamber on the bottom and to enable sensor readings, activation of another burn wire releases the benthic chamber lids;
6) Activation of the stirring paddles allows for mixing the seawater in the chambers;
7) The 8 pairs of syringes of the 2 VAMPIRES are activated by user-programmable times;
8) Finally, the ballast weights are released by the last burn wire and the lander floats back to the surface by virtue of its positive buoyancy.

### 2.6. Other Possible Configurations of the Amerigo Lander

The basic structure of the lander is the tripod, which is designed to land on the seabed by gravity, counteracted by the positive thrust of the buoys. It then performs its scheduled operations on the seabed and finally returns to the surface by virtue of positive buoyancy, after the release of the ballast weights. All the on-board instruments and devices are built to operate at depths up to 6000 m.

This setup makes the lander a vector that can host different types of instrumentation, such as sensors and probes for monitoring chemical and physical-chemical environmental parameters (pH, Eh, conductivity, temperature, salinity (calculated), oxygen, methane, pCO_2_, H_2_S) in the water column during its descent, its permanence on the bottom, and its ascent. 

The lander has also been designed to host instruments such as a microprofiler, to study sediment-water interface properties, a penetrometer, to measure the mechanical properties of surface sediments, a gravimeter, to measure seismicity on the seafloor, a corer, to collect sediment cores for early diagenesis, pollution, stratigraphy, or other studies, and passive samplers of water column and sediment solutes, to study pollution and environmental processes and to determine background values. In any case, the open architecture of the electronics allows for fitting other instruments and performing other operations.

Finally, it is a modular device, consisting of the buoy array, the recovery pole, the ballast weights, and the removable and replaceable instruments that perform different tasks in different environments, from very shallow waters, like lagoons and salt marshes, to shelf areas and abyssal planes.

### 2.7. The Automatic Benthic Chamber

**Figure 14:**
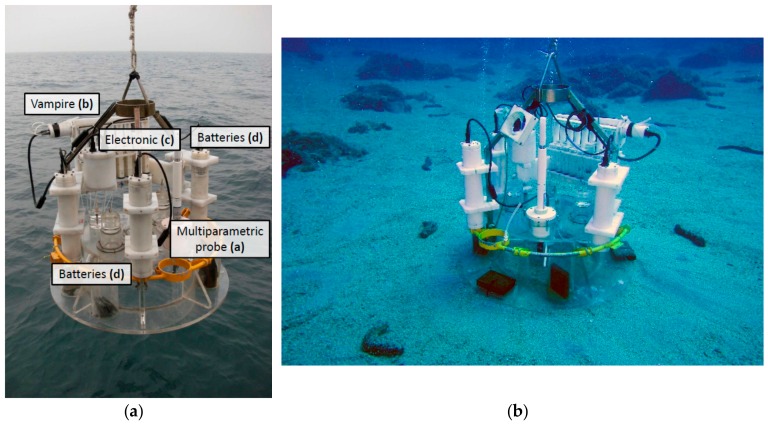
The Automatic Benthic Chamber during (**a**) deployment and (**b**) operational on the seabed with various instruments: Multiparameter probe; VAMPIRE; electronics case; and battery pack cases.

The CBA (Figure 14) has been developed as an alternative to the Amerigo Lander for missions involving measurements in shallow and transitional waters or work that needs to be carried out quickly and economically. This is made possible by the fact that the CBA does not require expensive maintenance, it is practical and fast to fit out, it is light and easy to maneuver, and it is deployed and recovered simply with a rope, which means that it can also be managed by small vessels.

The CBA can also be mounted on the Amerigo Lander, instead of the three benthic chambers, when measurements of benthic fluxes of dissolved chemical species are to be performed over a wider area. 

The present CBA is an automated device based on earlier manual benthic chambers managed by divers [60,61]. It is a Plexiglas cylinder open on the bottom and closed on top, which confines a known volume of water (approximately 100 L) overlying a known sediment area (3116 cm^2^) (Figure 14b). Its inner diameter is 63 cm and its height is 30 cm, of which 5 cm penetrate into the sediment and 25 cm remain above it, due to a lateral horizontal fin (Figure 14). The CBA is fitted with two valves on its top side, to let out the water entering the chamber during descent and landing (Figure 14). Like the Amerigo Lander, the CBA is equipped with an internal stirring system that reproduces the hydrodynamics near the seabed, which is responsible for the formation of the benthic boundary diffusion layer and for the intensity of the dissolved fluxes in the benthic chamber. The stirring system consists of a four-arm rotating paddle fitted on top of the inner side of the chamber. The paddle is actioned by the coupling of an electric motor (CBF Motors SRL, CRB35GM, CBF motors srl, Lissone, Italy) with a Neodymium magnet (Supermagnete, Magnetic disk 30 mm in diameter, 15 mm in height, Neodymium, N42, nickel-plated, Webcraft GmbH, Gottmadingen, Deutschland ) and turns at a speed of 4–6 rpm. In the CBA, this motor is activated immediately before deployment by connecting directly the batteries to the motor.

The CBA is also equipped with a multiparameter probe (Hydrolab MS5, OTT HydroMet, Kempten, Germany) for continuous monitoring of temperature, pH, conductivity, dissolved oxygen, Eh, and salinity (calculated) in the chamber (Figure 14). Like the Amerigo Lander, it is also fitted with the VAMPIRE system for collecting water samples inside and outside the chamber and for injecting tracers inside the chamber at programmable times. The motor of the VAMPIRE is activated by simple, easily programmable, and commercially available electronics (Idec MicroSmart FC6A PLC, IDEC Corporation, Sunnyvale, CA, USA). The cases housing the electronics, the battery packs, and the motors driving the VAMPIRE and the stirring paddle are made in Delrin and are built to withstand hydrostatic pressure up to a depth of about 200 m. Additionally, in the CBA, the VAMPIRE motor and the electronics cases are pressure-compensated by a silicone tube system filled with a non-conductive liquid (simple Vaseline oil), which affords resistance to high water pressures. 

The power supply of the CBA consists of three battery packs (NI-MH size D, 12 V, 8 Ah, Torricella SRL, Milano, Italy) housed in cylindrical Delrin cases (Figure 14). Two packs are connected directly to the electronics that supply and manage the VAMPIRE motor and one pack is connected directly to the rotating paddle, while the multiparameter probe has its own power supply system.

As regards the planning of CBA operations, the syringe sampling time is set by programming the electronics, while the probe measurement time is programmed by the software of the probe itself.

With regard to deployment and recovery, the CBA is deployed on the seabed and recovered by a rope which, during measurement activities, is attached to and marked by a buoy and a light.

The CBA, both in the standalone configuration and installed in the Amerigo Lander, is a low-cost device that does not require divers or connection cables to the support ship, thus saving the steep cost of divers and the technical problems posed by the connection cable. Further savings are afforded by the fact that the electronics and the batteries are commercially available, hence the low-cost. 

Like the Amerigo Lander, the CBA has a temperature operating limit which coincides with the lowest operating value of the sensors fitted in the benthic chamber and the polycarbonate, which is 50 °C (Hydrolab MS5 Multiprobe). However, if the CBA is used without the multiparameter probe the value is <140 °C (polycarbonate).

## 3. Benthic Flux Calculation

The benthic chambers of the Amerigo Lander and the CBA have been designed to measure the release/adsorption of dissolved substances at the sediment-water interface. The principle of their measurement with benthic chambers involves establishing the concentration differences of a solute over time in a known volume, confined over a known area of sediment [62].

Basically, the benthic fluxes of dissolved substances in each benthic chamber of the lander and of the CBA are calculated (2) by dividing the concentration of each solute, measured in the samples collected in the chamber by the syringes—typically nutrients such as ammonium, nitrites, nitrates, phosphates and silica, carbonate species (DIC, alkalinity, pCO_2_), trace elements (heavy metals), and organic pollutants—or recorded by the sensors (oxygen, methane, and pH), at the time of the collection or measurement, multiplied by the volume of the benthic chamber and divided by its base area [44].

(2) $$Di=\frac{\partial C_{i}}{\partial t}V/A,$$

where *Di* is the flux of solute *i*, *C_i_* is the concentration of chemical *i*, *t* is the time of sample collection or sensor measurement, *V* and *A* are the real volume and the area of each benthic chamber. 

**Figure 15:**
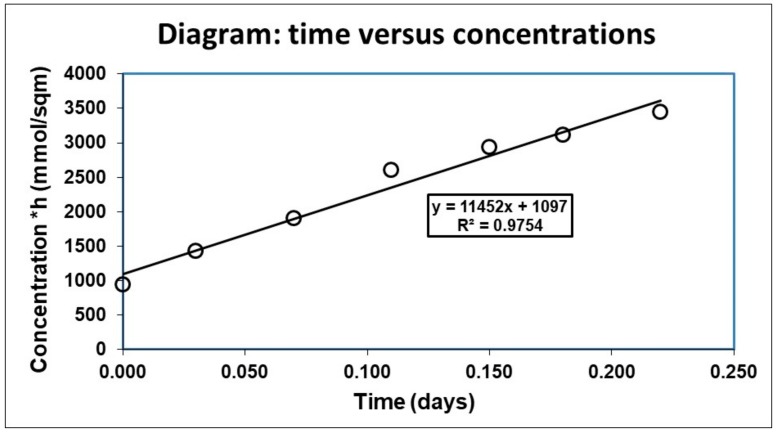
Diagram illustrating the time of water sample collection in the benthic chamber against its concentration and the correlation line.

In practice, the benthic fluxes of each solute are computed as Equation (3) by multiplying the slope of the line, calculated by a least square fit with time (days) on the x-axis and the concentration at different times, multiplied by the height of the benthic chamber, on the y-axis (Figure 15).

(3) $$D_{i}=y_{i},$$

where *y_i_* is the slope of the time vs. the concentration line of Figure 15.

During deployment, the real volume of each chamber is determined by injecting a solution of a non-reactive solute (tracer), e.g., CsCl, BrCl or deionized water, at a known concentration into the chambers [44] and subsequently measuring its concentration in the water samples collected in the syringes as Equation (4).

(4) $$V_{2}=\frac{V_{1}*C_{1}}{C_{2}},$$

where V_2_ is the real volume of the benthic chamber, C_1_ is the tracer concentration in the syringe, V_1_ is the volume of the tracer injected into the chamber, and C_2_ is the tracer concentration in the chamber after the injection. 

Theoretically, the tracer concentration in the chamber after the initial injection should be constant. If this does not happen, there are three possible explanations, as follows: The benthic chamber is not well placed on the bottom, there are leaks, or an irrigation process is under way in the bottom sediment.

## 4. Discussion

The Amerigo Lander [63,64,65,66,67,68,69,70,71] and the CBA [41,42,70,71,72,73,74,75,76,77] have successfully been tested and used in measurement and research activities carried out in the framework of international and national projects and in environmental investigations into the impacts of human activities on marine (e.g., harbor sediment dredging) or land environments (e.g., quality of drinking water). 

The Amerigo Lander has been tested and employed in shallow, medium, and deep-sea environments, whereas the CBA has been used up to a depth of 140 m. 

**Figure 16:**
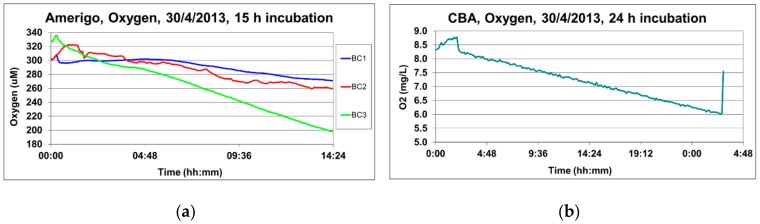
(**a**) Oxygen values recorded in the three benthic chambers of the Amerigo Lander (BC1, BC2, BC3) during deployment; (**b**) oxygen values recorded in the CBA during deployment of the two devices at the same time and site, a mud bottom sediment rich in fresh organic matter (Po River Prodelta).

As a discussion of the data collected by the two devices, we report (Figure 16) the trend of the dissolved oxygen concentrations, measured in the benthic chambers of the lander (by the AANDERAA optode oxygen sensors, Aanderaa Data Instruments AS, Bergen, Norway) and the CBA (by the Hydrolab MS5 oxygen sensor, OTT HydroMet, Kempten, Germany), at the same deployment time and site [71], and on a pelitic and organic matter-rich bottom in front of the Po River Estuary [78,79]. All the benthic chambers of the two devices recorded similar continuously decreasing values, due to mineralization of the high content in fresh reactive organic matter, deposited in front of the Po River Estuary. 

**Figure 17:**
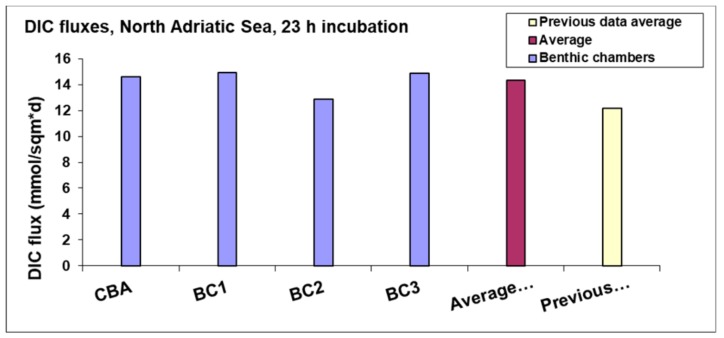
DIC fluxes measured by the CBA, by the Amerigo Lander (BC1, BC2, BC3), and in previous investigations at the same site, a mud bottom sediment rich in fresh organic matter (Po River Prodelta).

The benthic fluxes of solutes, whose concentrations were determined in the water samples collected by the VAMPIRE syringes, also showed reliable data. In fact, very similar values were determined for the fluxes of dissolved inorganic carbon (DIC) (Figure 17) measured by the three benthic chambers of the Amerigo Lander and by the CBA, deployed at the same and site, i.e., on pelitic and fresh organic matter-rich bottom sediments. Furthermore, these DIC flux values are very similar to those measured in earlier studies using different benthic chamber devices [80,81,82] at the same site and in the same season (Figure 17). 

On the whole, the oxygen and DIC data reported above (and other solute flux data that are not shown but are available from the authors) demonstrate that the Amerigo Lander and the CBA provide very similar information on benthic fluxes of dissolved substances and that these data are comparable with flux information recorded in previous work conducted at the same site. These first data, therefore, provide very good support for the correct functioning of both our devices.

The Amerigo Lander and the CBA record oxygen concentrations in the different deployment phases. In particular, the oxygen sensors can monitor the oxygen concentrations inside the chambers, the oxygen fluxes at the sediment-water interface can be calculated, and, furthermore, the oxygen trend can be used to check the closing of the benthic chamber.

**Figure 18:**
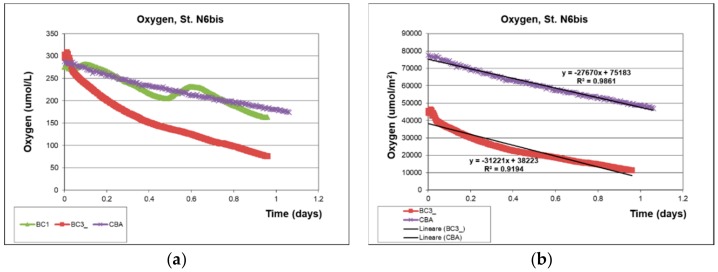
(**a**) Oxygen concentrations measured in the CBA and in two benthic chambers (BC1 and BC3) of the Amerigo Lander at the same site, a mud bottom sediment rich in fresh organic matter (Po River Prodelta); (**b**) multiplication of the oxygen concentrations by the height of the benthic chamber of the CBA and in BC3 against time (days) allows calculating the benthic fluxes (slope of the regression line (black line of Figure 18b)).

The trends of the oxygen concentrations recorded in the CBA and in two benthic chambers of the Amerigo Lander (BC1 and BC3) is shown in Figure 18. The data refer to the same time and station, on pelitic bottom sediment in front of the Po River Estuary.

The declining oxygen concentration in the CBA and in the Ox3 chamber, due to benthic respiration or sediment-water interface fluxes, can be appreciated in Figure 18a. Notably, the two peaks in the oxygen concentration trend in the Ox1 chamber of the lander demonstrate that the lid opened twice. 

In Figure 18b, the oxygen concentrations were multiplied by the height of the benthic chambers calculated by the dilution of the Cs tracer (4). The oxygen flux was then obtained by the slope of the regression line between time (days) and concentration (µmol/m^2^) (black line, Figure 18b).

**Figure 19:**
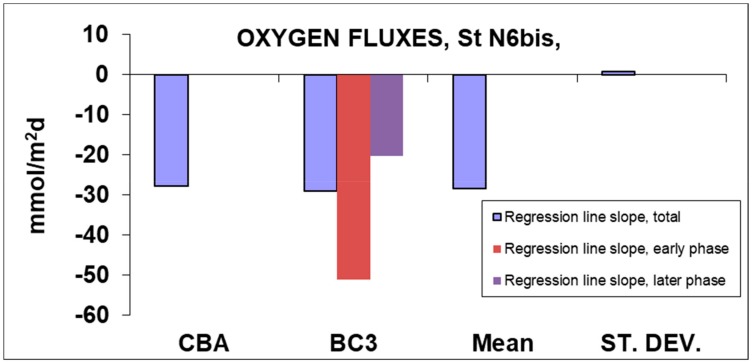
Oxygen fluxes calculated based on the data collected in the CBA and in the BC3 chamber of the Amerigo Lander. Deployment in front of the Po River Prodelta.

The fluxes calculated by the slope of the regression line are shown in Figure 19 and Table 2. As demonstrated by the examination of Figure 18, the flux measured in the CBA is almost constant over the 24 h incubation. For this reason, only the total flux was calculated (Figure 19 and Table 2). In contrast, the oxygen concentration trend in BC3 shows a decreasing flux that can be divided into early (with higher values) and later (with lower values). This is due to the small size of the chamber of the lander, which is more responsive to changes in the environmental conditions inside the chamber, like the reduction in fresh reactive organic matter and oxygen consumption.

**Figure 20:**
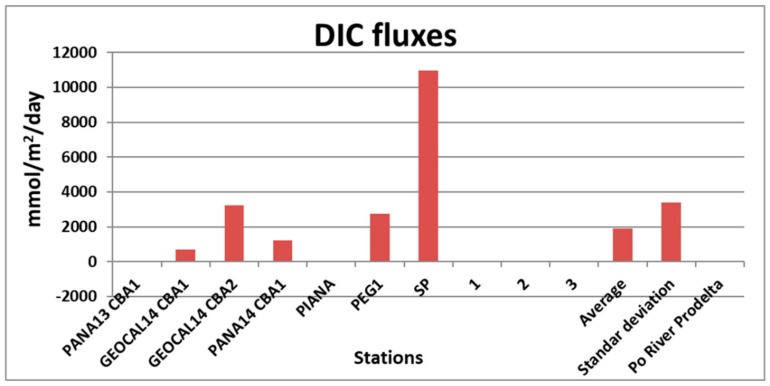
DIC fluxes measured by the CBA in the submarine volcanic area of Panarea and in front of the Po River Prodelta.

The CBA has also been deployed in a volcanic environment, at multiple sites on the seabed around the volcanic complex of Panarea, to measure the dissolved fluxes of DIC and metals released from the bottom in the gas vent area [39,43,44,54]. Figure 20 and Table 3 demonstrate the marked difference in DIC fluxes on the bottom between sites affected by vent fluxes (GEOCAL14CBA1, GEOCAL14CBA2, PANA14CBA1, PEG1, SP) and sites devoid of fluxes of dissolved substances at the sediment-water interface (PANA13CBA1, PIANA, 1, 2, 3), due to a surface layer of iron oxyhydroxide [39]. In addition, Figure 20 and Table 3 show very different DIC fluxes on the seafloor around the Panarea volcanic area, which is involved by vent fluxes, and the average DIC benthic fluxes measured in front of the Po River Estuary.

## 5. Conclusions

The Amerigo Lander and the CBA, two new instruments for measuring the benthic fluxes of dissolved substances, built by the authors, are presented herein. Both devices are autonomous and can operate in shelf (CBA) and deep-sea (Amerigo Lander) environments. The Amerigo Lander can also be used for other investigations of shallow and deep benthic ecosystems, because it can carry several different instruments. Both devices have been successfully tested and employed in international and national research projects and in environmental investigations of anthropic impacts by local authorities. These tests and activities have demonstrated the sound performance of the Amerigo Lander and the CBA, as also reflected by the DIC and oxygen data reported above. The CBA has also proved suitable for deployment in a volcanic area affected by gas and fluid vents, for which very few data are available due to technical measurement difficulties. In case of use in volcanic environments, the temperature of the solutes released from the bottom should be carefully monitored because of the temperature operation limits of the sensors (40–50 °C) or of the polycarbonate (140 °C).

Notably, these new instruments mark an important advancement in the Italian marine technology community, providing the means to compete for international research and applicative projects at the same level as foreign institutions.

## Figures and Tables

**Table 1:** Technical specifications of the sensors installed in the Amerigo Lander.

Sensor Type	Company	Parameter	Resolution	Initial Accuracy	Maximum Depth (m)
SBE37-SI (CTD)	Sea Bird Electronics	Conductivity (S/m)	0.00001	0.0003	7000
		Temperature (°C)	0.002	0.0001	7000
		Depth (m)	0.002% FS	0.1% FS	7000
SBE5T (Pump)	Sea Bird Electronics				7000
Oxygen Optode 3830	AANDERAA	Oxygen concentration (µM)	<1 µM	<8 µM	6000
		Air saturation (%)	0.4%	<5%	6000
Seapoint Turbidity Meter	Seapoint	Turbidity (FTU)	10 mV/FTU (range 500 FTU)	<1%	6000
Mets	Franatech	Methane	2.44 µM (range 50 nM–10 µM)	<1%	4000
pH	AMT	pH	0.01	0.05	6000

**Table 2:** Values of the oxygen fluxes at the sediment-water interface, calculated by the regression of all points (total), the phase with higher slope (early phase) and the lower slope (later phase).

.	Regression Line Slope, Total	Regression Line Slope, Early Phase	Regression Line Slope, Later Phase
**Oxygen**	mmol/m2*days		
**CBA**	−27.670	-	-
**BC3**	−28.903	−51.087	−20.144
**Mean**	−28.287	-	-
**ST. DEV.**	0.872	-	-

**Table 3:** Values of the DIC fluxes at the sediment-water interface measured by the CBA in the submarine volcanic area of Panarea and in front of the Po River Prodelta.

Cruise	Stations	Flux (mmol/m^2^·d)
PANA13	PANA13 CBA1	60.60
GEOCAL14	GEOCAL14 CBA1	689.30
GEOCAL14	GEOCAL14 CBA2	3223.90
PANA14	PANA14 CBA1	1212.70
PANA15	PIANA	−17.99
PANA15	PEG1	2750.60
PANA15	SP	10978.00
PANA15B	1	61.54
PANA15B	2	−4.41
PANA15B	3	−19.29
	-	-
Average	-	1893.49
Standard deviation	-	3408.76
Po River Prodelta average	-	29.00

## Appendix A

**Table A1:** Main configuration of the Amerigo Lander electronic.

Type	Description
Microprocessor	ATmega128
	RAM flash 128 Kb
	RAM 4Kb
	e2prom 4Kb
	Internal clock
	n. 3 timer 16 bit
	n. 8 ADC channels 10 bit
	n. 2 TTL serial ports
External circuit	serial flash RAM 4Mb
	n. 2 RS232 serial ports
	n. 3 RS485 ports
	n. 8 analogic inputs n. 2 digital inputsn. 20 on/off ports (H-bridge with electronic shunt)

**Table A2:** Configurations of the general inputs and outputs of the electronic of the Amerigo Lander.

Name	Type	Description
OUT 1	ON/OFF	Power terminals for CTD sensor
OUT 2	ON/OFF	Power terminals for O2 sensor number 1, chamber n.1
OUT 2	ON/OFF	Power terminals for O2 sensor number 2, chamber n.2
OUT 4	ON/OFF	Power terminals for O2 sensor number 3, chamber n.3
OUT 5	ON/OFF	Power terminals for CH4 sensor number 1, chamber n.1
OUT 6	ON/OFF	Power terminals for CH4 sensor number 2, chamber n.2
OUT 7	ON/OFF	Power terminals for CH4 sensor number 3, chamber n.3
OUT 8	ON/OFF	Power terminals for analogic sensors (n.2 turbidity and n.1pH)
OUT 9	ON/OFF	Power terminals - Engine palette chamber n.1 (mixing water)
OUT 10	ON/OFF	Power terminals - Engine palette chamber n.2 (mixing water)
OUT 11	ON/OFF	Power terminals -Engine palette chamber n.3 (mixing water)
OUT 12	ON/OFF	Engine VAMPIRONE number 1
OUT 13	ON/OFF	Engine VAMPIRONE number 2
OUT 14	ON/OFF	Burn wire n.1 Buoy electronic check
OUT 15	ON/OFF	Burn wire n.2 Benthic chamber release
OUT 16	ON/OFF	Burn wire n.3 Benthic chamber cover release
OUT 17	ON/OFF	Burn wire n.4 Benthic chamber cover release
OUT 18	ON/OFF	Burn wire n.5 weights release
OUT 19	ON/OFF	Power terminals 24 Volts for microprofiler
OUT 20	ON/OFF	OxyStat pump
RS232 1	RS232	Main serial port - PC communication
RS232 2-1	RS232	CTD
RS232 2-2	RS232	serial communication with O2 sensor n. 1, chamber n.1
RS232 2-3	RS232	serial communication with O2 sensor n. 2, chamber n.2
RS232 2-4	RS232	serial communication with O2 sensor n. 3, chamber n.3
RS232 2-5	RS232	serial communication with pH sensor n. 1, chamber n.1
RS232 2-6	RS232	serial communication with pH sensor n. 2, chamber n.2
RS232 2-7	RS232	serial communication with pH sensor n. 3, chamber n.3
RS485 1	RS485	serial communication with CH4 sensor n. 1, chamber n.1
RS485 2	RS485	serial communication with CH4 sensor n. 2, chamber n.2
RS485 3	RS485	serial communication with CH4 sensor n. 3, chamber n.3
an 1	analogical in	Analogical (0-5V) Turbidity sensor n. 1, chamber n.1
an 2	analogical in	Analogical (0-5V) Turbidity sensor n. 2, chamber n.2
an 3	analogical in	Analogical (0-5V) Turbidity sensor n. 3, chamber n.3
an 4	analogical in	NC-future purpose (pCO_2_)
an 5	analogical in	NC-future purpose (Eh)
an 6	analogical in	NC-future purpose (H_2_S)
an 7	analogical in	NC-future purpose (other sensor)
an 8	analogical in	NC-future purpose (other sensor)
DI 1	digital IN	magnetic sensor (future purpose, VAMPIRONE position)
DI 2	digital IN	magnetic sensor (future purpose, VAMPIRONE position)
